# Effect of Aging Treatment on the Damping Capacity and Mechanical Properties of Mg-6Al-1Zn Alloy

**DOI:** 10.1155/2015/170458

**Published:** 2015-03-31

**Authors:** Abdel-Wahab El-Morsy, Ahmed I. Z. Farahat

**Affiliations:** ^1^Mechanical Engineering Department, Faculty of Engineering-Rabigh, King Abdulaziz University, P.O. Box 344, Rabigh 21911, Saudi Arabia; ^2^Mechanical Engineering Department, Faculty of Engineering-Helwan, Helwan University, 1st Sherif Street, Helwan, Cairo 11792, Egypt; ^3^Plastic Deformation Department, Central Metallurgical Research and Development Institute, 1st Elfelezat Street, El-Tebbin, Helwan 12422, Egypt

## Abstract

The damping capacity and mechanical properties of Mg-6Al-1Zn alloy after heat treatment were investigated. The damping characteristics of un-heat-treated, solution treated, and aged Mg-6Al-1Zn specimens were determined by measuring the damping ratio and the logarithmic decrement of free vibrations of a bending beam clamped at one side. The microstructural evaluations confirmed that the *β*-Mg_17_Al_12_ phase was reprecipitated after aging and increased with an increase in aging time. The peak level of damping ratio and logarithmic decrement was obtained after 34 hr of aging time, over which the damping capacity declined according to increasing amount of strong pining points.

## 1. Introduction

Magnesium alloys have great potential for the applications in suppressing mechanical vibration and attenuating wave propagation for the control of noise and the stabilization of structure components because of their low density and good damping capacity [1–4]. However, the use of magnesium alloys has been limited to narrow range because of the limited mechanical properties such as low hardness and low tensile strength [5]. Recently, a large number of research studies have focused on improving the mechanical properties of magnesium alloys by alloying [6, 7], plastic deformation [8–12], or heat treatment [9, 13]. However, the improvement of mechanical properties often results in a decline of damping capacity [14, 15]. For further engineering applications of magnesium, balancing the damping behavior and the mechanical properties has been a critical challenge [14, 16].

The damping capacity of magnesium alloys is considered to be associated with different characteristics of precipitation, such as the presence and interaction of precipitation, and crystal lattice defects, such as dislocations and impurity atoms [17–19]. A good combination of mechanical properties and damping capacity could be achieved if the dislocations are firmly pinned by the impurity atoms and the phase precipitates, but on the other hand the dislocations are allowed to vibrate between these pinning points [20–22].

In the present work, the effect of heat treatment (solid solution and different aging durations) on the internal damping capacity and mechanical properties of Mg-6Al-1Zn (commercially known as AZ61) alloy and their relations was investigated. The damping tests were conducted using impulse-frequency response method with single cantilever vibration mode at room temperature. The intrinsic damping behavior was discussed in light of the microstructure characteristic and damping data.

## 2. Material and Experimental Procedure

**Figure 1:**
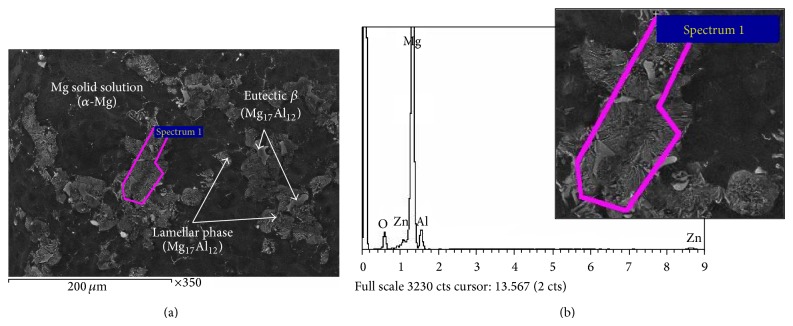
SEM micrograph (a) and EDS (b) of un-heat-treated AZ61 Mg-alloy.

The experiments were conducted using Mg-6Al-1Zn alloy (AZ61). The chemical composition of the used material is listed in Table 1.  Figure 1 shows the SEM micrograph of un-heat-treated (as-received) AZ61 alloy specimen. The un-heat-treated specimen shows a lamellar mixture of depleted Mg solid solution (*α*-Mg) and intermetallic Mg_17_Al_12_ compound. The intermetallic compound is revealed to be a mixture of discontinuous and continuous Mg_17_Al_12_ *β*-phase at grain boundaries [23]. The EDS of the un-heat-treated specimen shown in Figure 1(b) reveal that the elemental compositions of both gray and bright phases include Mg, Zn, O and Al.

**Figure 2:**
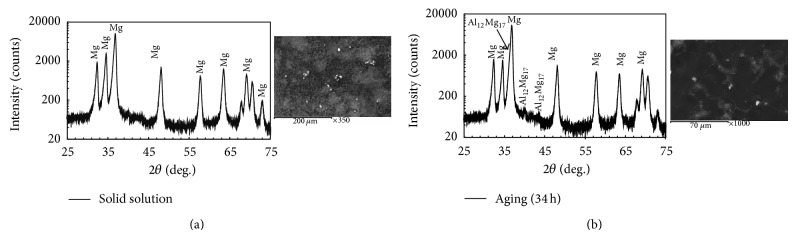
X-ray diffraction patterns of (a) solution treated and (b) aged AZ61 alloy for 34 hr.

The un-heat-treated specimens were subjected to solid solution treatment at 410°C for 24 hr followed by water quenching. The *β*-phase and eutectic phase (formed at the grain boundaries during the casting into the matrix) disappeared and the matrix transformed to single phase, supersaturated solid solution. The specimens were aged at 200°C afterwards, for different intervals of time. The X-ray diffraction pattern of the solution treated and aged for 34 hr is shown in Figure 2. The heat treatment of AZ61 alloy is fundamentally a microstructure transformation process in which Mg_17_Al_12_ phase dissolves during solid solution and then reprecipitates during aging and increases with the increase in aging time [24].

**Figure 3:**
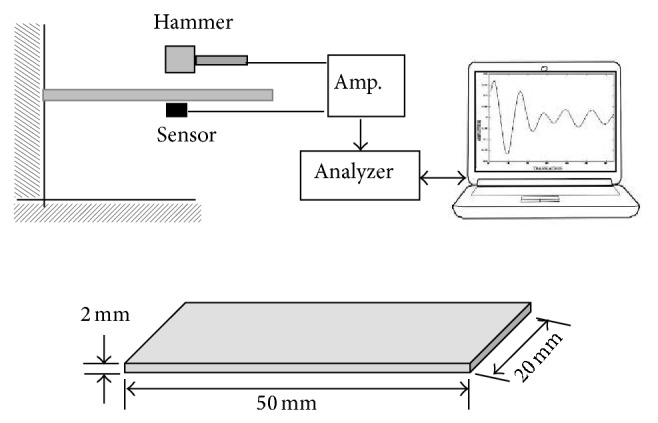
Schematic diagrams of experimental setup for the damping test.

Damping test was performed using impulse-frequency response method with single cantilever vibration mode, which can simply and rapidly measure damping property of the material. The testing schematic diagram is shown in Figure 3. The dimensions of the damping test specimens were 50 mm × 20 mm × 2 mm. The damping test was conducted at room temperature. The measurement sequence consists of recording the free vibrations of the specimens excited by knocking it with an adequate hammer. After the exciting hammer hits the specimen, the amplitude of vibration gradually decreases with time as the vibration energy is dissipated. These decayed amplitudes and frequency of vibration were transferred to oscilloscope and computer by an accelerometer attached on the specimen. The amplitude decay as a function of time and the vibration modes was detected by the acquisition data system.

Several techniques are used to quantify the level of damping in a vibration structure [25–27]. Frequency domain approach (half-power bandwidth method) was used in this work as it is most commonly used to measure damping ratio in frequency domain [28]. This method is based on the frequency response function curve and consists in obtaining the maximum amplitude related to the natural frequency of the system and the two frequencies that have response amplitude equal to the maximum divided by the square root of two points corresponding to half power point. The loss factor *η* of this method can be defined as

(1) $$\begin{array}{c} \eta =\frac{\omega_{2}-\omega_{1}}{\omega_{n}}=\frac{\Delta \omega }{\omega_{n}}, \end{array}$$

where *ω*_*n*_ is the frequency at which the peak of the curve is obtained and *ω*_1_ and *ω*_2_ are the two frequencies, for which the frequency response is 1/√2 times the one at resonance. The damping ratio *ξ* in a single degree of freedom is given by

(2) $$\begin{array}{c} \xi =\frac{\eta }{2}. \end{array}$$

For small damping (*ξ* ≪ 1), the logarithmic decrement, *δ*, is defined as

(3) $$\begin{array}{c} \delta =\pi Q^{-1}, \end{array}$$

where *Q* ^−1^ is the internal friction and can be calculated from the bandwidth Δ*ω* by

(4) $$\begin{array}{c} Q^{-1}=\frac{\omega_{2}-\omega_{1}}{\omega_{n}}=\frac{\Delta \omega }{\omega_{n}}. \end{array}$$

## 3. Results and Discussion

**Figure 4:**
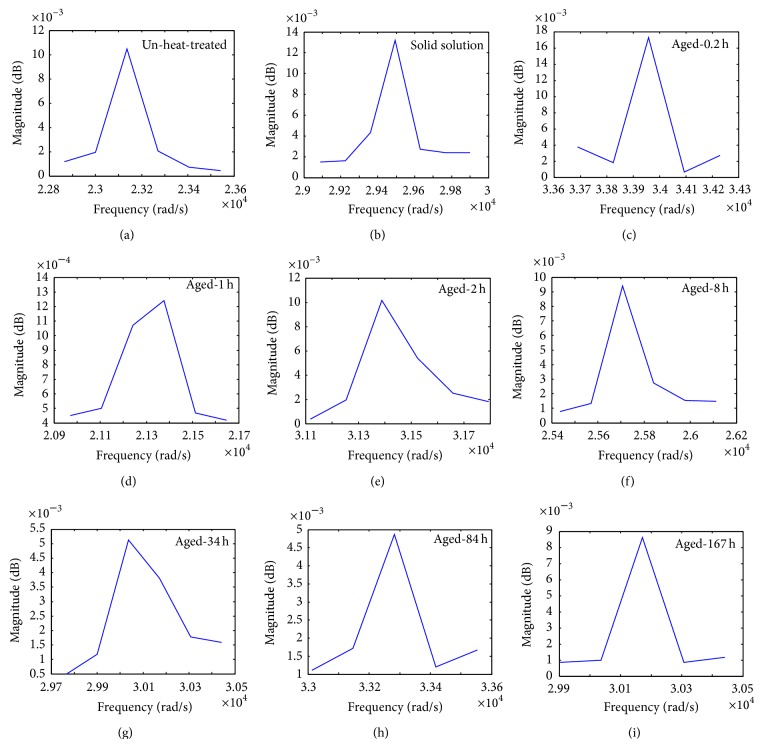
Frequency response variation as a function of frequency transformation using MatLab program.

Figure 4 shows frequency response variation as a function of frequency transformation using calculations carried out in MatLab program. The signals input into fast Fourier transform (FFT) were settled by Fourier transformation using MatLab program to measure the damping capacity from damping test of cantilever. The resonant frequencies of the un-heat-treated, solution treated, and aged AZ61 at 200°C for different intervals of time were calculated from these curves and listed in Table 2. For a quantitative measure of damping behavior, the half-power bandwidth method was used. The frequency response function is plotted by linear curve fitting as in the Figure 4, and the resonant frequency (*ω*_*n*_) and half-power bandwidth frequency (Δ*ω*) are calculated and damping loss factor (*η*) can be determined using the data in (1). The Δ*ω* was determined from the resonant peak value.

The usual convention is to consider points *ω*_1_ and *ω*_2_ to be located at frequencies on the response curve where the amplitude of response of these points is 1/√2 times the maximum amplitude. The bandwidth at these points is frequently referred to as half-power bandwidth. Mandal et al. [25] reported that half-power bandwidth method is useful for only small loss factor and the thickness of the specimen must be smaller than the corresponding wavelength.

**Figure 5:**
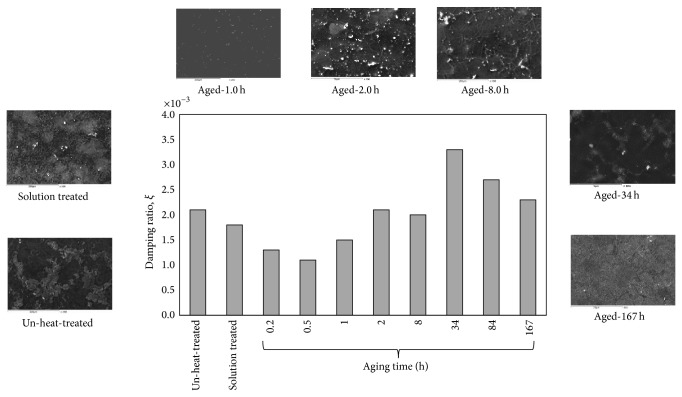
The corresponding damping ratio as a function of the aging time, compared with the un-heat-treatment and solid solution treated specimens.

Figure 5 illustrates the corresponding damping ratio, *ξ*, of the AZ61 as a function of the aging time at 200°C, compared with the un-heat-treated and solution treated specimens. The un-heat treated specimen has 2.58 × 10^−3^ damping ratio. After solid solution, the damping ratio value slightly decreases to 2.24 × 10^−3^, which is resulted due to dissolution of the *β*-Mg_17_Al_12_ phase. However, after aging, the damping ratio continuously decreases to approximately 0.5 hr of aging time. An increasing trend is observed in the damping ratio up to 34 hr aging time, after the damping ratio shows a reduction. The peak drop of 1.8 × 10^−3^ is observed at 167 hr aging time.

**Figure 6:**
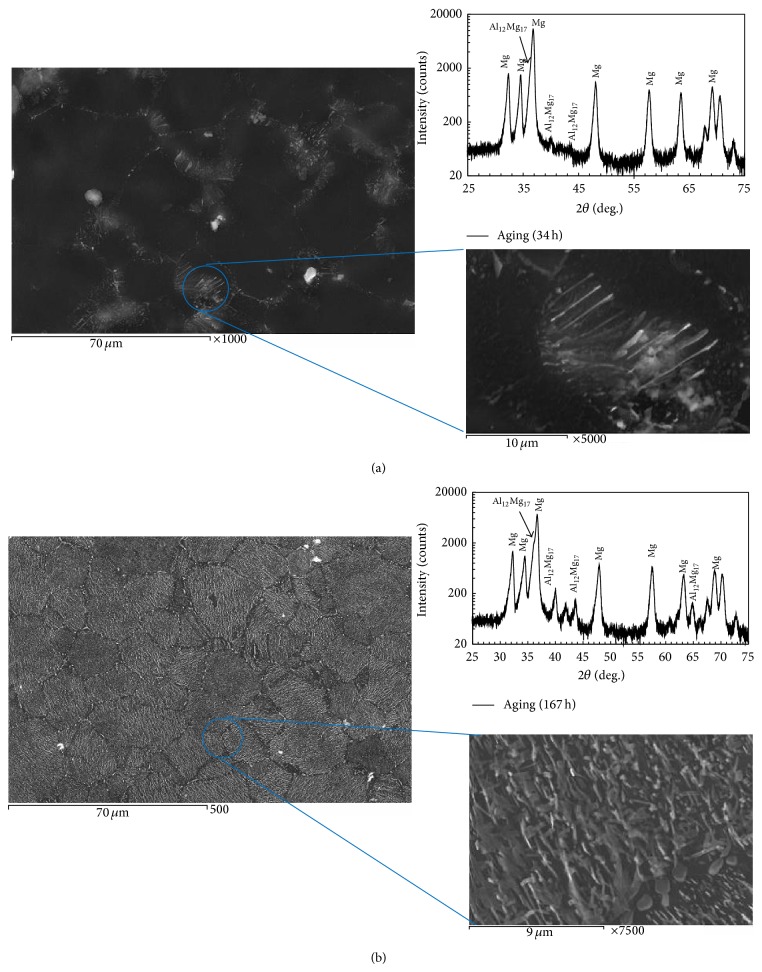
SEM micrograph of aged AZ61 alloy (a) for 34 hr and (b) for 167 hr aging times.

The damping ratio of the specimen aged at 34 hr increased because of increasing the interface area of the two phases with different stiffness, which is caused by discontinuous *β*-phase precipitation inside *α*-Mg matrix. Xu et al. [29] confirmed that numerous discontinuous Mg_17_Al_12_ were distributed at the newly formed dynamic recrystallized grain boundaries as shown in Figures 6(a) and 6(b). Enhancement of damping properties is recognized to be attained by dislocation, interface of particles with different stiffness, and high damping particle or phase.

The decline in damping ratio after 34 hr aging time is appraised to be caused by the hardening of microstructure coinciding with the transition from *α*-Mg matrix with discontinuous *β*-phase to lamellar structure (Figure 6(b)). The SEM micrograph shown in Figure 6(a) reveals that the alloy exhibits some Mg_17_Al_12_ precipitates at the grain boundaries and becomes interconnected to form a network. As it can be seen from Figure 6(b), there is substantive lamellar *β*-phase distributing in the microstructure. The lamellar structure which is the product of the discontinuous precipitation always initiates at grain boundaries (in Figure 6(a)) and grows perpendicular to the boundary. The previous studies observed that the lamellar structures appear mostly under 200°C and it is hard to see them above 250°C [30]. The damping ratio of specimen aged at 167 hr aging time decreased by about 30% after formation of lamellar structure.

**Figure 7:**
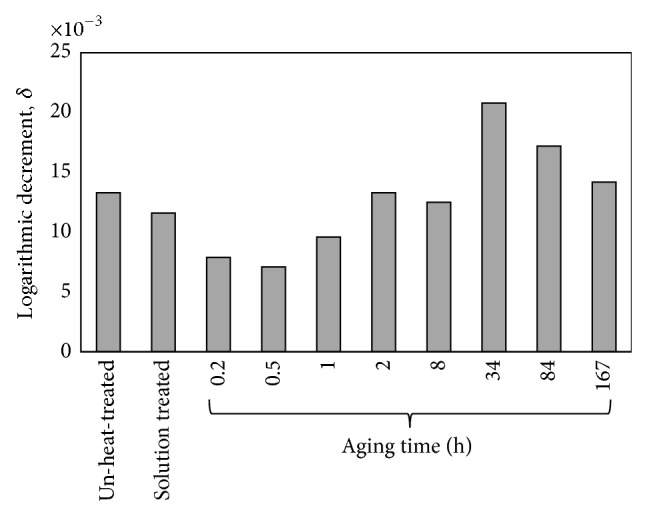
The logarithmic decrement of un-heat-treated, solution treated, and aged AZ61 alloy.

Figure 7 illustrates the logarithmic decrement, *δ*, of the un-heat-treated, solution treated, and aged AZ61. The figure describes the effect of the Mg_17_Al_12_ precipitates on the decay property. It has already been observed from the SEM investigation that, after solid solution, the *α*-Mg solution becomes in the supersaturated state and thereby there will be a number of solute atoms distributing along the grain boundaries that play a pinning role, which makes the movement of grain boundaries more difficult and thus the decay is decreased. After aging treatment up to 34 hr aging time, most of the solute Al atoms precipitate in the *β*-phase and decrease the concentration of solute Al atoms in the *α*-Mg matrix and the spacing between weak pining points becomes large. Thus, the diffusion of Mg atoms along grain boundaries becomes smooth and consequently the logarithmic decrement after aging process increases, as shown in Figure 7. At longer aging time (>34 hr) the precipitation hardening which is based on the principle of restricting dislocation mobility generally leads to reduction in the decay values.

**Figure 8:**
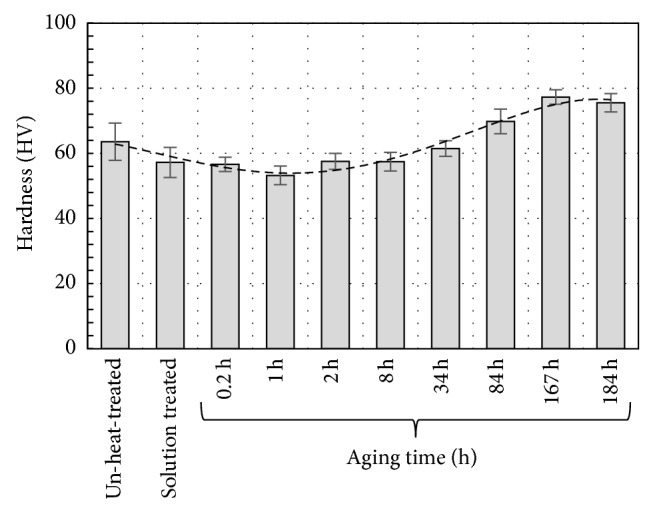
Measured hardness values of AZ61 alloy.

Figure 8 illustrates the corresponding hardness measurements (HV) of the AZ61 alloy. The un-heat-treated specimen has hardness value of 64 HV. After 24 hr solid solution treatment, the hardness value decreases to 57 HV. The small deviations exhibited by the un-treated and the solution treated specimens are assumed to be arisen from the fact that the un-treated specimen has already some *β*-phase. However, the Figure shows a slight decrease in the hardness value in the initial stages of aging treatment up to approximately 2 hr, which may be associated with a decrease in the dislocation density due to recovery and grain boundary relaxation processes.

After prolonged aging time up to 34 hr, it is clearly noticed that the HV values are slightly increased. Thereafter, with further increase in the aging time up to approximately 167 hr, the dependence of hardness values on the aging time became more obvious. The increase in hardness values is likely to be due to the precipitation of *β*-phase in the matrix. By extending aging time (184 hr), the effect of grain growth becomes more dominant than the strengthening effect due to *β*-precipitation [13].

**Figure 9:**
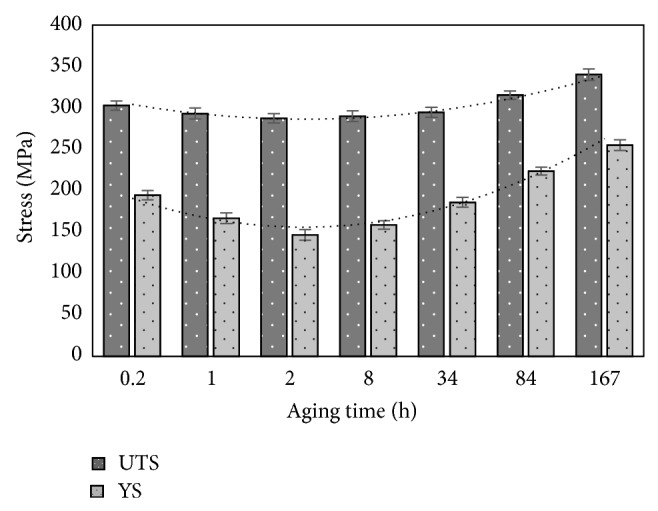
Measured tensile properties of AZ61 alloy with different aging time.

Figure 9 shows the tensile properties curves at ambient temperature for the AZ61 alloy aged at 200°C. At the beginning of aging (up to 2 hr), the tensile strength and the yield stress slightly decreased with aging time. When being aged up to 34 hr, all samples have shown slightly increased strength value. When aging time exceeds 34 hr, the tensile strength value increases due to the development of Mg_17_Al_12_ precipitates

## 4. Conclusions 

The damping behavior of heat treated AZ61 alloy has been investigated. The following have been found.

- Upon aging for 2 hr there is a reappearance of the discontinuous precipitation (*β*-phase Mg_17_Al_12_), with the lamellar structure of *α*-Mg. At 167 hr aging time, and the majority of grains are totally transformed into lamellar structure.
- On increasing the aging time up to 34 hr, the dependence of damping ratio on the aging time became more pronounced because of the precipitation of *β*-phase. Longer aging time (>34 hr) introduces higher amounts of Mg_17_Al_12_ precipitates in the microstructure that are able to pin the dislocations as strong pinning points, which in turn cause lower damping values.
- The hardness values and the tensile strength of the aged AZ61 alloy are decreased to approximately 2 hr of aging time. After prolonged aging time up to 34 hr, the alloy started to show slight increase in mechanical properties values. When being aged for 167 hr, the dependence of mechanical properties on the aging time becomes more obvious.

## Figures and Tables

**Table 1:** Chemical composition of Mg-alloy AZ61 (% weight).

Al	Cr	Mn	Si	Fe	Zn	Mg
6.7	0.012	0.25	0.047	0.001	1.11	Balance

**Table 2:** Resonant frequencies of AZ61 alloy.

	*A*_max⁡_ (×10^−3^)	$A_{\max}/\sqrt{2}$ (×10^−3^)	*ω*_1_	*ω*_2_	*ω*_*n*_	*ξ* (×10^−3^)	*Q* ^−1^ (×10^−3^)	*δ* (×10^−3^)
AC	10.5	7.40	3674.4	3690.0	3682.2	2.1	4.2	13.3
SS	13.2	9.30	4684.9	4702.2	4694.2	1.8	3.7	11.6
0.2 hr	17.3	12.2	5397.8	5419.8	5404.8	1.3	2.5	7.90
0.5 hr	14.8	10.5	6281.1	6295.4	6287.7	1.1	2.3	7.10
1 hr	6.40	4.50	5376.1	5392.5	5383.3	1.5	3.1	9.6
2 hr	10.2	7.20	4987.9	5009.1	4995.7	2.1	4.2	13.3
8 hr	9.40	6.60	4084.0	4100.2	4091.3	2.0	4.0	12.5
34 hr	5.10	3.60	4772.2	4803.8	4780.4	3.3	6.6	20.8
84 hr	4.90	3.40	5287.4	5316.4	5297.2	2.7	5.5	17.2
167 hr	8.60	6.10	4794.8	4816.5	4801.9	2.3	4.5	14.2

## References

[1] Nishiyama K., Matsui R., Ikeda Y., Niwa S., Sakaguchi T. (2003). Damping properties of a sintered Mg-Cu-Mn alloy. *Journal of Alloys and Compounds*.

[2] Schaller R. (2003). Metal matrix composites, a smart choice for high damping materials. *Journal of Alloys and Compounds*.

[3] Gentle C. R., Lacey M. R. (1999). Design of a novel insulated construction material. *Materials and Design*.

[4] Friedrich H., Schumann S. (2001). Research for a ‘new age of magnesium’ in the automotive industry. *Journal of Materials Processing Technology*.

[5] Diqing W., Jincheng W., Gaifang W. (2008). Effect of Mn on damping capacities, mechanical properties, and corrosion behaviour of high damping Mg–3 wt.%Ni based alloy. *Materials Science and Engineering A*.

[6] Alam M. E., Hamouda A. M. S., Nguyen Q. B., Gupta M. (2013). Improving microstructural and mechanical response of new AZ41 and AZ51 magnesium alloys through simultaneous addition of nano-sized Al_2_O_3_ particulates and Ca. *Journal of Alloys and Compounds*.

[7] Suresh K., Rao K. P., Prasad Y. V. R. K., Hort N., Kainer K. U. (2013). Microstructure and mechanical properties of as-cast Mg-Sn-Ca alloys and effect of alloying elements. *Transactions of Nonferrous Metals Society of China*.

[8] El-Morsy A., Ismail A., Waly M. (2008). Microstructural and mechanical properties evolution of magnesium AZ61 alloy processed through a combination of extrusion and thermomechanical processes. *Materials Science and Engineering A*.

[9] Volkov A., Kliukin I. (2015). Improving the mechanical properties of pure magnesium through cold hydrostatic extrusion and low-temperature annealing. *Materials Science and Engineering: A*.

[10] Zhang H., Yan Y., Fan J. (2014). Improved mechanical properties of AZ31 magnesium alloy plates by pre-rolling followed by warm compression. *Materials Science and Engineering A*.

[11] Song B., Xin R., Chen G., Zhang X., Liu Q. (2012). Improving tensile and compressive properties of magnesium alloy plates by pre-cold rolling. *Scripta Materialia*.

[12] Diez M., Kim H., Serebryany V., Dobatkin S., Estrin Y. (2014). Improving the mechanical properties of pure magnesium by three-roll planetary milling. *Materials Science and Engineering: A*.

[13] El-Morsy A., Farahat A. (2012). The influence of age hardening on the microstructure and mechanical behavior of wrought magnesium alloy AZ61. *Steel Research International*.

[14] Wan D., Wang J., Yang G. (2009). A study of the effect of Y on the mechanical properties, damping properties of high damping Mg-0.6%Zr based alloys. *Materials Science and Engineering A*.

[15] Hu X.-S., He X.-D., Zheng M.-Y., Wu K. (2010). Effect of small tensile deformation on damping capacities of Mg-1% Al alloy. *Transactions of Nonferrous Metals Society of China (English Edition)*.

[16] Wang J., Wei W., Huang X., Li L., Pan F. (2011). Preparation and properties of Mg–Cu–Mn–Zn–Y damping magnesium alloy. *Materials Science and Engineering A*.

[17] Lihua L., Xiuqin Z., Xianfeng L., Haowei W., Naiheng M. (2007). Effect of silicon on damping capacities of pure magnesium and magnesium alloys. *Materials Letters*.

[18] Feng S., Zhang W., Zhang Y., Guan J., Xu Y. (2014). Microstructure, mechanical properties and damping capacity of heat-treated Mg-Zn-Y-Nd-Zr alloy. *Materials Science and Engineering A*.

[19] Yan B., Dong X., Ma R., Chen S., Pan Z., Ling H. (2014). Effects of heat treatment on microstructure, mechanical properties and damping capacity of Mg-Zn-Y-Zr alloy. *Materials Science and Engineering A*.

[20] Hu X.-S., Wang X.-J., He X.-D., Wu K., Zheng M.-Y. (2012). Low frequency damping capacities of commercial pure magnesium. *Transactions of Nonferrous Metals Society of China*.

[21] Hu X. S., Zhang Y. K., Zheng M. Y., Wu K. (2005). A study of damping capacities in pure Mg and Mg-Ni alloys. *Scripta Materialia*.

[22] Hu X. S., Wu K., Zheng M. Y., Gan W. M., Wang X. J. (2007). Low frequency damping capacities and mechanical properties of Mg-Si alloys. *Materials Science and Engineering: A*.

[23] Wen C., Yamada Y., Shimojima K., Mordike B., Kainer K. (2000). Microstructure evolution and mechanical properties of AZ91 Mg foams. *Proceedings of the 5th International Conference on Magnesium Alloys and their Applications, Munich, Germany*.

[24] Zhang Z., Zeng X., Ding W. (2005). The influence of heat treatment on damping response of AZ91D magnesium alloy. *Materials Science and Engineering A*.

[25] Mandal N. K., Rahman R. A., Leong M. S. (2004). Experimental study on loss factor for corrugated plates by bandwidth method. *Ocean Engineering*.

[26] Umashankar K. S., Abhinav A., Gangadharan K. V., Vijay D. (2009). Damping behaviour of cast and sintered aluminium. *ARPN Journal of Engineering and Applied Sciences*.

[27] Rivière A. (2003). Measurement of high damping: techniques and analysis. *Journal of Alloys and Compounds*.

[28] Bertha A., Roesset J. M. Analytical evaluation of the accuracy of the half-power bandwidth method to estimate damping ratios in a structure.

[29] Xu S. W., Matsumoto N., Kamado S., Honma T., Kojima Y. (2009). Effect of pre-aging treatment on microstructure and mechanical properties of hot compressed Mg-9Al-1Zn alloy. *Materials Science and Engineering A*.

[30] Lai W.-J., Li Y.-Y., Hsu Y.-F., Trong S., Wang W.-H. (2009). Aging behaviour and precipitate morphologies in Mg-7.7Al-0.5Zn-0.3Mn (wt.%) alloy. *Journal of Alloys and Compounds*.

